# Profiling Fecal Microbiome and Metabolites in a Feline Infectious Peritonitis Virus Challenge Model in Kittens: A Pilot Discovery Study

**DOI:** 10.3390/ijms27177843

**Published:** 2026-09-02

**Authors:** Shuye Zhen, Hong Yue, Yajie Pan, Ping Mu, Ruitong Zhang, Jiaqi Wei, Ziyan Meng, Yang Liu, Jianlong Zhang, Linlin Jiang, Jiayu Yu, Xin Yu, Xingxiao Zhang

**Affiliations:** 1 School of Life Sciences, Ludong University, Yantai 264025, China; 2 Yantai Key Laboratory of Animal Pathogenetic Microbiology and Immunology, Yantai 264025, China; 3 Provincial Engineering Research Center for Pet Animal Vaccines, Yantai 264025, China; 4 Collaborative Innovation Center for the Pet Infectious Diseases and Public Health in the Middle and Lower Stream Regions of the Yellow River, Yantai 264025, China; 5 College of Veterinary Medicine, Shandong Agricultural University, Tai’an 271017, China

**Keywords:** feline infectious peritonitis (FIP), feline infectious peritonitis virus (FIPV), fecal microbiome, metabolites

## Abstract

Feline infectious peritonitis (FIP), caused by FIPV, is a highly fatal disease. The coordinated shifts in gut microbiota, metabolome, and their interactions following FIPV infection remain poorly understood. In this pilot discovery work, we established an oral FIPV DF-2 challenge model in kittens, which reproduced typical FIP phenotypes confirmed by clinical scores and pathology for exploratory multi-omics profiling. Using V3–V4 16S rRNA sequencing on Illumina PE300 and UHPLC-MS-based untargeted metabolomics, combined with LEfSe, OPLS-DA, and Pearson correlation, we preliminarily characterized fecal microbial and metabolic alterations associated with FIP onset in this exploratory animal model. FIP-affected kittens exhibited reduced abundances of *Ligilactobacillus*, *Bifidobacterium*, and *Limosilactobacillus*, alongside elevated *Roseburia*, *Helicobacter*, and *Campylobacter*. Differential metabolites were enriched in six core pathways, which preliminarily suggest potential links to oxidative stress, energy metabolism impairment, immune suppression, and systemic inflammatory response during FIP progression. Correlation analyses linked beneficial genera to upregulated 4-hydroxynonenal and xanthosine, while opportunistic taxa (*Helicobacter*, *Campylobacter*, *Enterobacterales*) correlated with caffeine, 1-methyluric acid, and retinoic acid derivatives. These preliminary findings suggest unique fecal microbial-metabolic signatures in FIP, providing hypothesis-generating mechanistic insights and potential therapeutic targets. However, given the exploratory nature of this pilot study, these results require validation in larger, independently controlled cohorts.

## 1. Introduction

Feline infectious peritonitis (FIP), induced by the feline infectious peritonitis virus (FIPV), represents a lethal systemic condition in cats, marked by immune-mediated vascular inflammation and a high mortality rate [1,2]. This disease is typified by aberrant activation of the host’s immune system, resulting in the deposition of immune complexes, both localized and systemic inflammatory responses, and subsequent damage to multiple organs, notably the liver, spleen, kidneys, and peritoneum [3,4]. FIP presents as two distinct clinical phenotypes: effusive (“wet”) FIP, distinguished by body cavity effusion, and noneffusive (“dry”) FIP, characterized by pyogranulomatous lesions. Despite advances in understanding the pathogenic mechanisms of FIPV, the development of effective therapeutic interventions remains a significant challenge.

Recent research has highlighted the pivotal role of gut microbiota in regulating host immune responses, metabolic functions, and disease progression. An imbalance in the composition or function of the gut microbiota, known as dysbiosis, has been linked to a variety of immune-mediated diseases, including allergies, autoimmune disorders, and inflammatory bowel disease, among others [5]. Furthermore, several existing studies have preliminarily confirmed alterations in feline intestinal flora under FCoV infection [6], yet most only detected single-layer microbial shifts without supporting metabolomic data. Previous studies have demonstrated that intestinal microbiota influences host immune responses and energy metabolism by modulating metabolites such as short-chain fatty acids, amino acids, and lipids [7,8]. It is essential to characterize fecal microecology and metabolic reprogramming during FIPV infection for disease research: gut microecology directly mediates intestinal barrier function and systemic immune homeostasis, and microbial-derived metabolites act as key signaling molecules bridging virus infection and inflammatory lesions. Current studies still have three prominent gaps: first, lacking a natural oral FIPV kitten model to compare healthy baseline and clinically diseased individuals; second, missing integrated microbiome–metabolome correlation analysis to decode microbe–metabolite interactive signatures; third, no reports clarify core perturbed metabolic pathways and their pathological relevance to FIP progression. Moreover, these findings may suggest novel strategies for the prevention and treatment of FIP, such as enhancing the immune response and metabolic health of cats through modulation of the intestinal microbiota, thereby alleviating the pathogenic effects of FIPV.

In this study, an oral FIPV DF-2 (GenBank accession number: JQ408981.1) challenge model was developed in kittens. The microbiome and metabolome of fecal samples from kittens in both healthy states and after FIPV infection were analyzed. Consequently, the abundance of *Ligilactobacillus*, *Bifidobacterium*, and *Limosilactobacillus* was reduced in FIP-affected kittens, whereas the prevalence of *Roseburia*, *Helicobacter*, and *Campylobacter* was increased. Furthermore, the differential metabolites were implicated in six significantly altered metabolic pathways, including caffeine metabolism, pyruvate metabolism, folate biosynthesis, and Th17 cell differentiation. These metabolites were also closely associated with specific differential genera.

Building on these exploratory multi-omics data from our pilot oral FIPV kitten model, this work screens candidate metabolic perturbations potentially linked to FIP progression in kittens, characterized by upregulated lipid peroxidation byproducts (e.g., 4-hydroxynonenal) and purine metabolites (e.g., xanthosine), alongside downregulated caffeine and its catabolites, retinoic acid, and tetrahydrofolate derivatives. These preliminary observations provide tentative clues for metabolic disruptions that may participate in FIP pathogenesis and hypothesize candidate markers for follow-up diagnostic and therapeutic research. Nonetheless, future research involving larger cohorts and a variety of clinical conditions is essential to validate and refine these metabolic signatures as diagnostic biomarkers.

## 2. Results

### 2.1. FIPV DF-2-Infected Kittens Develop Typical FIP Characteristics

In this study, 6-week-old kittens were divided into three FIPV DF-2 inoculation groups (10^4^, 10^5^, 10^6^ TCID_50_/mL, 5 kittens per group) and one blank control group (3 uninoculated kittens) (Table 1). Clinical signs of kittens were continuously monitored for 45 days post-inoculation (dpi), and FIPV viral loads in anal swabs from 0 to 45 dpi were detected by RT-qPCR, with relevant data summarized in Supplementary Table S5. No FIPV nucleic acid was detected in the blank control group throughout the experiment, with all viral loads below the detection limit.

Viral shedding exhibited a significant dose-dependent pattern. The FIP incidence rates of the low-, medium- and high-dose groups were 40%, 60% and 80%, respectively (Table 1). Higher inoculation doses corresponded to higher peak viral loads, earlier shedding onset, and longer shedding duration. Viral shedding was only detectable at the early infection stage (2–30 dpi). Fecal viral loads dropped rapidly to undetectable levels after kittens developed severe FIP lesions such as ascites and granulomas, confirming no positive correlation between fecal viral shedding and systemic disease severity. Significant individual shedding heterogeneity was attributed to infection dose gradients, individual immune differences, and the tropism shift of FIPV from intestinal epithelial cells to macrophages.

FIPV DF-2 infection induced typical FIP clinical signs in kittens, dominated by fever and lymphocytopenia, along with weight loss, lethargy, vomiting, jaundice, thoracic/abdominal effusions, and granulomas. Weight loss was synchronized with high fever and severe lymphocytopenia, while granulomas were mainly observed in severely diseased kittens. These clinical manifestations are consistent with previous studies [2,9,10], confirming the reliability of the experimental model.

A 0–3 four-grade clinical scoring system based on previous studies [10,11,12,13] was established to quantify eight core symptoms, with cumulative scores applied to evaluate disease severity. Diseased kittens maintained cumulative clinical scores above 10 points with persistent severe FIP symptoms. In contrast, non-diseased kittens only had transient mild fever in the early infection stage without core FIP lesions, and their scores quickly returned to normal without exceeding the 10-point threshold throughout the trial. The mild clinical fluctuations of non-diseased kittens were positively correlated with inoculation doses.

**Figure 1:**
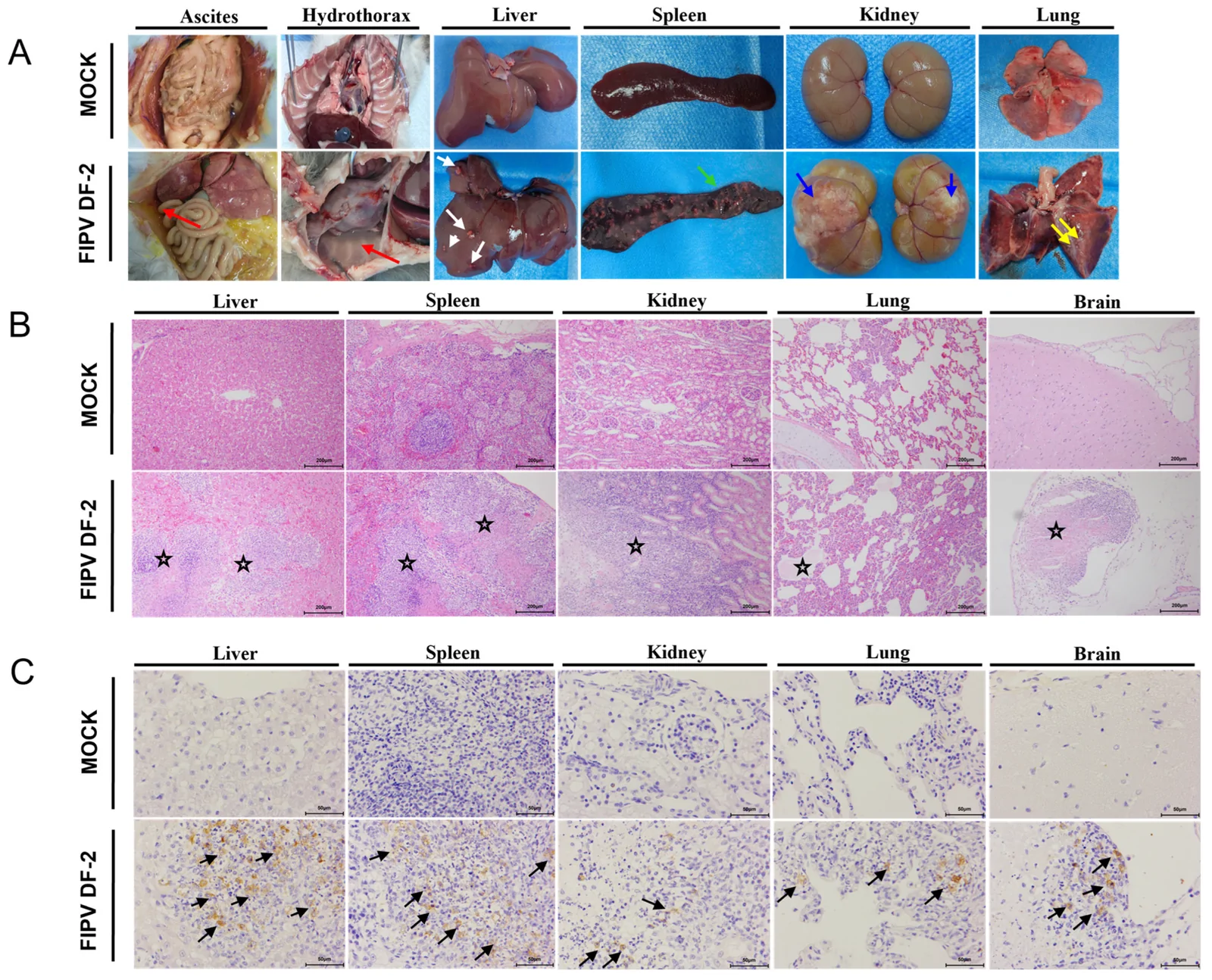
Necropsy and histopathology analysis of FIPV-infected and healthy cats. (**A**) FIPV DF-2: Gross necropsy of the cat euthanized with FIP. Postmortem examination of cats with FIP revealed straw-colored, protein-rich effusions in the abdominal and thoracic cavities (red arrows). The liver capsule contained raised and whitish foci, extending into the underlying parenchyma (white arrows). The serosal surface of the spleen was covered with punctate, coalescing fibrinous plaques (green arrow). An obvious granulomatous lesion (blue arrow) was observed on the capsule of the kidney, extending downward into the parenchyma. The lung capsule contained small punctate raised and whitish foci (yellow arrows). MOCK: Gross necropsy of the healthy cat. (**B**) HE-stained sections of different tissue sections, including the liver, spleen, kidney, lung, and brain. FIPV DF-2: In the liver, the liver cells showed diffuse severe degeneration and multifocal inflammatory necrosis; large tracts of spleen were necrotic with inflammatory cell infiltration. In the kidney, diffuse severe renal tubular degeneration, focal renal interstitial hemorrhage, mesangial matrix thickening, and other lesions were observed. In the lung, alveolar edema was accompanied by interstitial inflammatory cell infiltration. Focal necrosis of the meninges was observed in the brain, accompanied by inflammatory cell infiltration (black pentagrams). MOCK: Gross necropsy of the healthy cat. 100×, scale bar = 200 μm. (**C**) FIPV DF-2: Immunohistochemical assays showed specific positive signals for FIPV nuclear coat protein (N protein) detected in necrotic foci of FIPV-infected liver, spleen, kidneys, lungs, and brain tissue (black arrows). MOCK: Gross necropsy of the healthy cat. 100×, scale bar = 200 μm.

Histopathological and immunohistochemical findings, as shown in Figure 1, revealed typical FIP-associated pathological lesions in the affected kitten, including thoracic/abdominal protein-rich effusions, multi-organ granulomas, fibrin deposition, inflammatory necrosis, and interstitial infiltration in the liver, spleen, kidney, and lung. Organ lesions were directly caused by FIPV infection, while no pathological abnormalities were observed in the control group.

Based on comprehensive clinical, pathological, and immunohistochemical results, a cumulative clinical score > 10 points was defined as the diagnostic threshold for FIP onset, and fever combined with lymphocytopenia was identified as a simple diagnostic criterion without necropsy. According to the criteria, the overall FIP infection rate was 60% (9/15), and disease severity increased in a dose-dependent manner, with both wet and dry forms of typical FIP successfully induced. The ID_50_ of the DF-2 strain was calculated as 3.16 × 10^5^ TCID_50_ via the Reed–Muench method. This study successfully established a stable and reliable kitten FIP infection model using the FIPV DF-2 strain.

### 2.2. Differences in the Fecal Microbiome Associated with FIPV Infection

**Figure 2:**
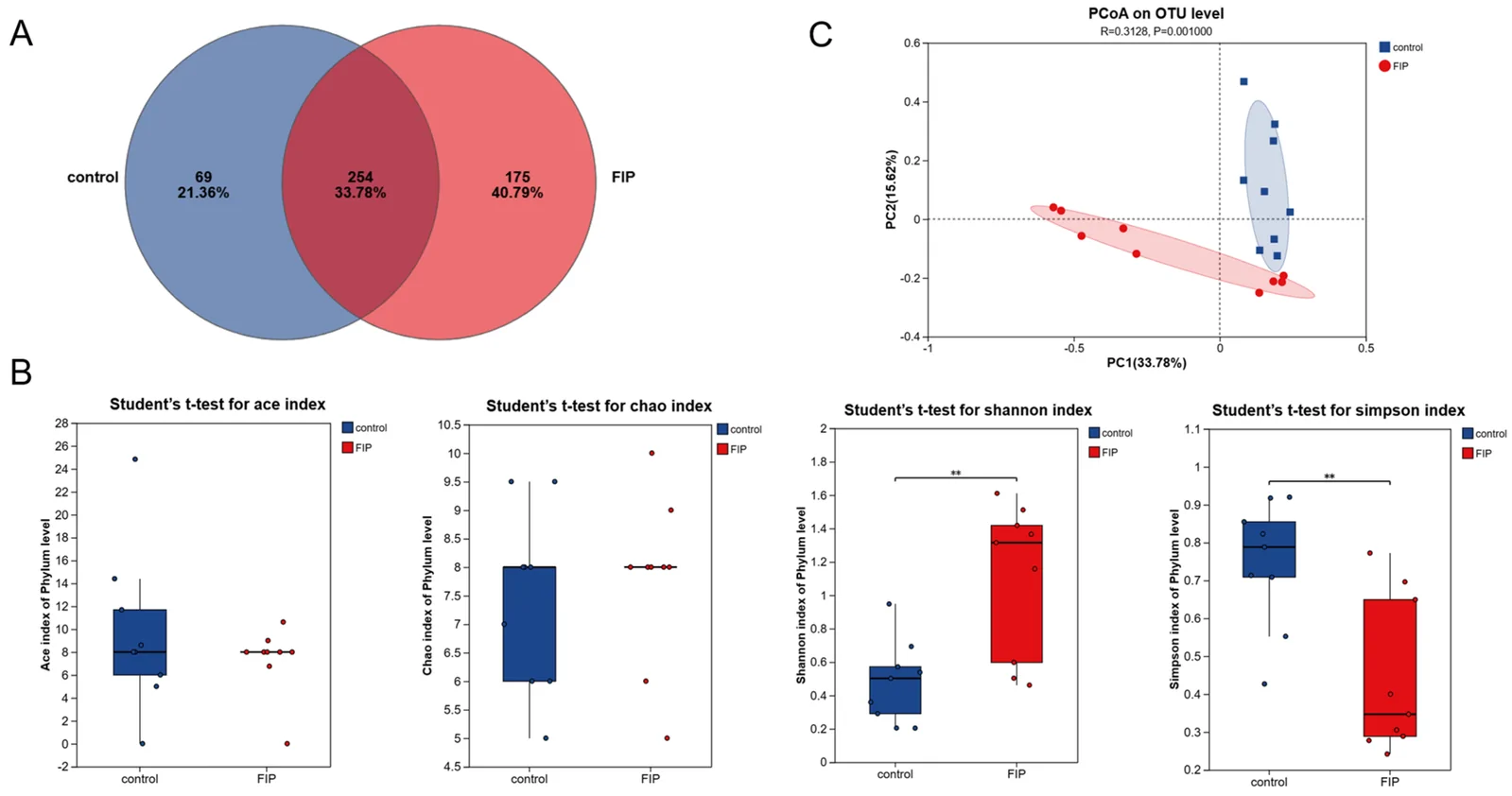
Fecal microbiota composition is altered in kittens with FIP. (**A**) Venn diagram displayed the shared and unique OTUs between the two groups. The total number of OTUs in the FIP group was much higher than that in the control group (429 OTUs versus 323 OTUs), while 254 OTUs were shared between the two groups. (**B**) Alpha diversity analysis of the fecal microbial community (Ace index and Chao index showed no significant difference in bacterial richness between the two groups, and Shannon index and Simpson index showed that the bacterial community diversity in FIP group was significantly higher than that in control group). (**C**) Plot of principal coordinate analysis (PCoA) of the fecal microbial community. The two different experiment groups were segregated into different community clusters.** *p* < 0.01, indicating significant differences.

Prior to integrated microbiome–metabolome comparative analysis, standardized sample filtering was performed to balance the sample size of two comparison cohorts. Finally, nine fecal samples from kittens with typical clinical FIP manifestations were defined as the FIP group, and nine pre-inoculation baseline fecal samples collected at day 0 from all experimental animals were selected as paired healthy control group, forming a balanced 9 vs. 9 sample set for multi-omics differential screening. Detailed sample information is provided in Supplementary Table S2. A total of 1,216,677 valid sequences were obtained for subsequent analysis, with an average of 67,593 sequences per sample (median: 66,942; range: 52,878–82,907). These sequences were clustered into 498 operational taxonomic units (OTUs) at a 97% similarity threshold using the UPARSE-OTU algorithm, forming the basis for subsequent microbiome diversity and compositional analyses. Both alpha and beta diversity analyses were performed to evaluate microbial richness, diversity, and community structure. Alpha diversity was assessed using the Ace and Chao indices for community richness, and the Shannon and Simpson indices for community diversity. As shown in Figure 2A, the FIP group presented more OTUs than the control group. No significant differences in Ace and Chao indices were observed between the two groups, indicating comparable microbial richness. However, the FIP group exhibited a significantly higher Shannon index and a lower Simpson index (Figure 2B), suggesting markedly increased fecal microbial diversity after FIPV infection. Beta diversity analysis further revealed distinct microbial community structures between the healthy and FIPV-infected kittens. Principal coordinate analysis (PCoA) based on Bray–Curtis distances at the OTU level showed clear separation of the two groups (Figure 2C), demonstrating that FIPV infection substantially alters fecal microbiome composition.

**Figure 3:**
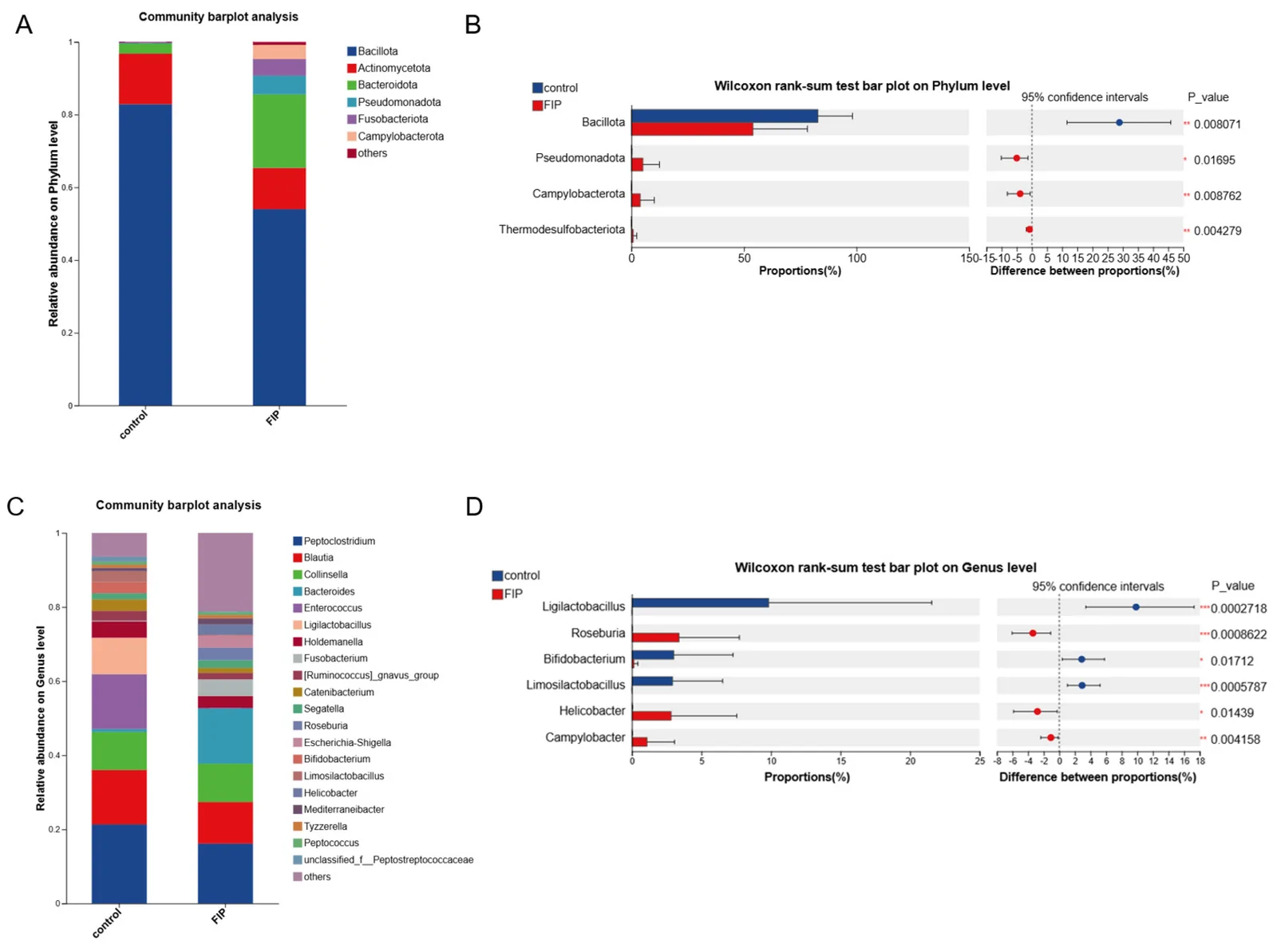

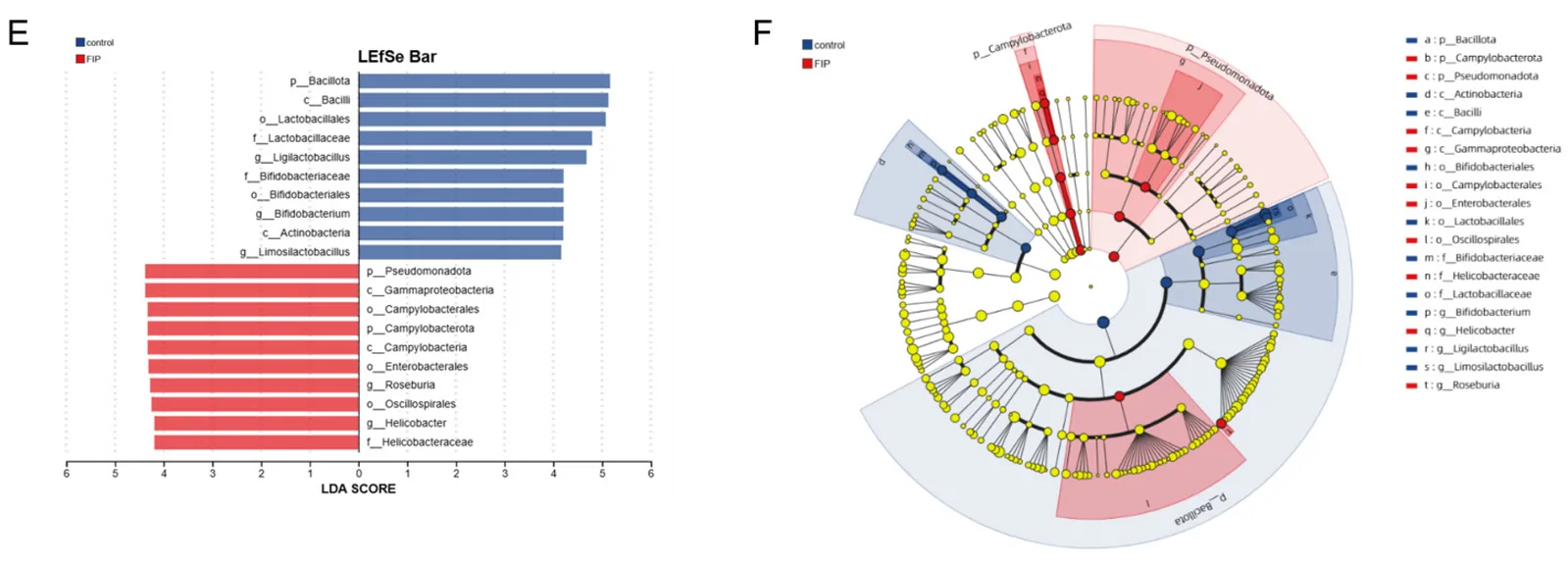
Altered fecal microbiota composition and biomarkers in FIP kittens. (**A**) Fecal bacterial community at the phylum levels. The relative abundance of the top 6 phyla of fecal microbiota in both healthy and FIP-affected kittens. The fecal samples from healthy and FIP-affected kittens are abbreviated as control and FIP, respectively. (**B**) Fecal bacterial community at the phylum level. The relative abundance of the top 20 genera of fecal microbiota in both healthy and FIP-affected kittens. (**C**) Differences in the relative abundance of bacterial genera linked to FIPV DF-2 infection. (**D**) Differences in the relative abundance of bacterial genera linked to FIPV DF-2 infection. (**E**) Linear discriminant effect size (LEfSe) analysis identified several bacterial taxa enriched in FIP-affected kittens (red) and controls (blue). (**F**) Cladogram reports the taxa showing different abundance values (LDA > 4) according to LEfSe. Yellow (nodes): Taxa with no significant difference between the two groups (non-discriminative taxa). Black (branches/connecting lines): Phylogenetic branches connecting taxa across taxonomic levels (phylum → class → order → family → genus). Light blue/light red background shading: Sector highlights marking the phylum−level clades where differentially abundant taxa are concentrated (blue = control−enriched phyla, red = FIP−enriched phyla). Statistical comparisons to the control group (C) are denoted as * *p* < 0.05, ** *p* < 0.01, *** *p* < 0.001,indicating significant differences.

Taxonomic annotation identified 12 bacterial phyla, among which Bacillota, Actinomycetota, and Bacteroidota dominated the fecal microbiota (Figure 3A). Compared with the control group, the FIP group showed enriched Bacteroidota and depleted Bacillota and Actinomycetota. Specifically, the relative abundances of *Pseudomonadota* (*p* = 0.01695), *Campylobacterota* (*p* = 0.008762), and *Thermodesulfobacteriota* (*p* = 0.004279) were significantly increased in the FIP group, while *Bacillota* was significantly decreased (*p* = 0.008071) (Figure 3B). At the genus level, six of the top twenty most abundant genera differed significantly between groups (Figure 3C). The FIP group exhibited significant upregulation of *Roseburia* (*p* = 0.0008622), *Helicobacter* (*p* = 0.01439), and *Campylobacter* (*p* = 0.004158), alongside significant downregulation of *Ligilactobacillus* (*p* = 0.0002718), *Bifidobacterium* (*p* = 0.01712), and *Limosilactobacillus* (*p* = 0.0005787) (Figure 3D).

Linear discriminant analysis effect size (LEfSe) was applied to screen potential microbial biomarkers, with a logarithmic LDA threshold of 4. A total of 20 differential taxa were identified, including 10 taxa enriched in the FIP group and 10 in the control group (Figure 3E). The cladogram indicated that the FIP group had higher abundances of Pseudomonadota-derived Gammaproteobacteria and *Enterobacterales*, as well as *Helicobacter*, *Oscillospiraceae*, and *Roseburia*. In contrast, the control group was characterized by enriched Bacillota-associated *Lactobacillales*, including *Ligilactobacillus* and *Limosilactobacillus* (Figure 3F).

### 2.3. Comparison of Metabolomic Profiling Between Healthy and FIPV-Infected Groups

**Figure 4:**
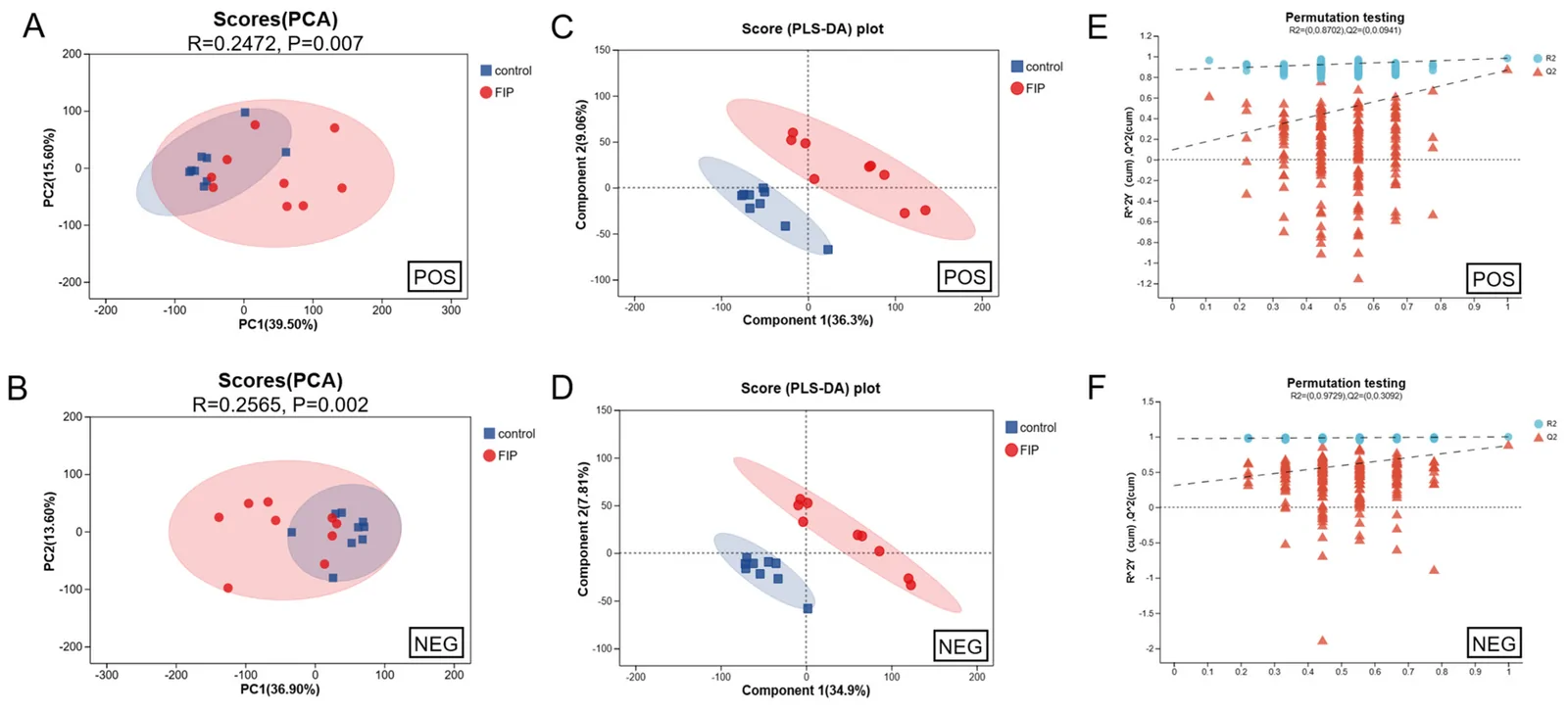
Fecal metabolomic profiles are distinct between FIP-affected and control kittens. (**A**,**B**) Principal component analysis (PCA) model. One point in the figure corresponded to one sample, blue represented the control group, and red represented the FIP group. The (**A**) is the PCA of feces in positive ion mode, the (**B**) is the PCA of feces in negative ion mode. (**C**,**D**) Scatterplot of fecal metabolite PLS-DA scores for control and FIP-affected groups. The C is the PLS-DA of feces in positive ion mode; the D is the PLS-DA of feces in negative ion mode. (**E**,**F**) PLS-DA replacement test, the E is in cationic mode, the F is in anionic mode. In PLS−DA score plots (**C**,**D**), dashed lines denote zero-reference axes for Component 1 (vertical) and Component 2 (horizontal). In permutation testing plots (**E**,**F**), the upper horizontal dashed line indicates the original R^2^Y value, the middle horizontal dashed line marks the Q^2^ = 0 baseline, and the diagonal dashed line represents the regression line of permuted Q^2^ values.

Consistent with the sample filtering standard applied in fecal microbiome analysis, we adopted a balanced 9 vs. 9 sample design for untargeted metabolomics comparison: nine samples from FIP-diseased kittens and nine pre-challenge healthy baseline samples. Principal Component Analysis (PCA) in both ion modes initially showed overlapping distributions, but ANOSIM confirmed significant separation (*p* < 0.05) between the groups (Figure 4A,B). While PCA is useful for visualizing sample distribution, it struggles to distinguish subtle group differences. Therefore, Partial Least Squares-Discriminant Analysis (PLS-DA) was employed, effectively highlighting metabolic pattern differences crucial for identifying FIP-related distinctions. Figure 4C shows significant separation in positive ion mode, with similar results in negative ion mode, consistent with positive ion findings (Figure 4D). The values for R2X, which quantify the proportion of variance in the independent variables accounted for by the model, and Q2, an indicator of the model’s predictive performance, together with the outcomes of permutation tests, confirmed the adequacy of the sample quality (Figure 4E,F). Collectively, these findings not only substantiate the significant metabolic disparities between the two groups but also affirm the effectiveness of the PLS-DA model in distinguishing between sample groups. This validation underscores the model’s predictive capability and its appropriateness for subsequent variance component analysis.

**Figure 5:**
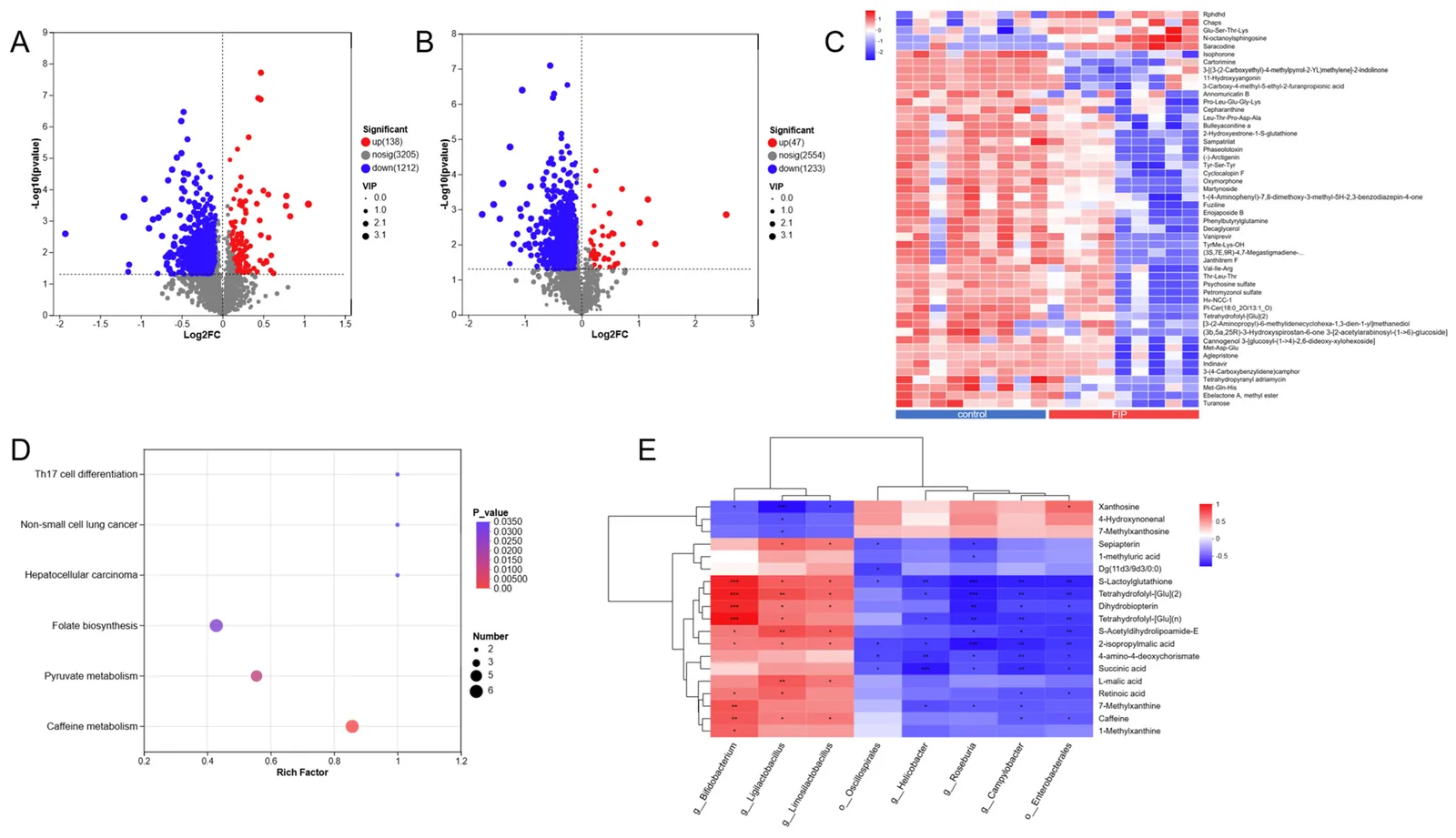
Differential fecal metabolites, enriched pathways, and metabolite–microbiota associations in FIP kittens. (**A**,**B**) Volcano plots of differential metabolites in FIP-affected versus control groups (VIP > 1, *p* < 0.05). Red dots denote up-regulated metabolites and blue dots denote down-regulated metabolites. Panel A shows data acquired in cationic mode, and panel B shows data acquired in anionic mode. (**C**) Heatmap of fecal differential metabolites (VIP > 1, *p* < 0.05, FC > 1.5) from FIP-affected and healthy control groups. Each column corresponds to one sample, and each row corresponds to one metabolite; color intensity reflects the relative abundance of each metabolite across samples. (**D**) Bubble diagram for metabolic pathway enrichment analysis between FIP-affected and healthy control groups. Circles represent matched metabolic pathways; circle color corresponds to the pathway *p*-value, and circle size corresponds to the pathway impact value. (**E**) Heatmap illustrating Pearson correlations between fecal metabolite clusters and fecal microbiota. Color gradients represent the magnitude of correlation coefficients; blue indicates negative correlation, and red indicates positive correlation. * *p* < 0.05, ** *p* < 0.01, *** *p* < 0.001 denote statistically significant correlations between metabolite clusters and fecal microbiota.

In this study, Variable Importance in the Projection (VIP) was employed as a crucial parameter for the identification of differential metabolites within the PLS-DA model, reflecting the significance of each variable. The default criteria for screening were established at VIP > 1 and *p* < 0.05, and the findings were illustrated using volcano plots (Figure 5A,B). After multivariate filtering via OPLS-DA and Benjamini–Hochberg FDR multiple comparison correction to exclude false-positive signals, a total of 2624 significantly differential metabolites were identified across positive and negative ion modes when comparing FIP-affected kittens to healthy controls, among which 184 metabolites were significantly upregulated and 2440 were significantly downregulated (Supplementary Table S3). All differential metabolites met the combined thresholds of VIP > 1, raw *p* < 0.05, and FDR-adjusted *p* < 0.05. Hierarchical cluster analysis of select differential metabolites (FC > 1.5) revealed a distinct separation between the fecal metabolic profiles of FIP-affected cats and healthy controls, underscoring significant disruptions in metabolic homeostasis linked to FIP pathogenesis (Figure 5C). Pathway enrichment analysis of the differential metabolites was conducted using the KEGG database. The significantly enriched KEGG pathways (*p* < 0.05) are presented as a bubble plot in Figure 5D, and the corresponding metabolite categories of the enriched pathways are summarized in Table 2. The findings suggest that the differential metabolites are predominantly involved in pathways related to caffeine metabolism, pyruvate metabolism, folate biosynthesis, hepatocellular carcinoma, non-small cell lung cancer, and Th17 cell differentiation. In total, six enriched pathways were associated with nineteen metabolites. Notably, 4-hydroxynonenal, 7-methylxanthosine, and xanthosine were significantly upregulated in the FIP group. Conversely, the concentrations of caffeine, 1-methyluric acid, S-acetyldihydrolipoamide-E, Dg(11d3/9d3/0:0), retinoic acid, 4-amino-4-deoxychorismate, 1-methylxanthine, dihydrobiopterin, 7-methylxanthine, 2-isopropylmalic acid, sepiapterin, tetrahydrofolyl-[Glu](n), S-lactoylglutathione, succinic acid, L-malic acid, and tetrahydrofolyl-[Glu](2) were significantly decreased in the FIP group (Supplementary Table S4).

### 2.4. Correlation Between the Altered Metabolites and Fecal Microbiome

To investigate the relationship between the fecal microbiome and the metabolome, Pearson correlation analysis was conducted on various fecal microbes and metabolites. The analysis revealed that genera with increased abundance in the FIP group exhibited a positive correlation with metabolites that were significantly upregulated in the FIP group, and a negative correlation with downregulated metabolites, and vice versa (Figure 5E). Notably, among the genera that were decreased in the FIP group, *Bifidobacterium* demonstrated a significant positive correlation with several metabolites, including S-lactoylglutathione, tetrahydrofolyl-[Glu](2), dihydrobiopterin, tetrahydrofolyl-[Glu](n), S-acetyldihydrolipoamide-E, 2-isopropylmalic acid, retinoic acid, 7-methylxanthine, caffeine, and 1-methylxanthine. Furthermore, *Ligilactobacillus* demonstrated a positive correlation with several metabolites, including sepiapterin, S-lactoylglutathione, tetrahydrofolyl-[Glu](2), dihydrobiopterin, tetrahydrofolyl-[Glu](n), S-acetyldihydrolipoamide-E, 2-isopropylmalic acid, L-malic acid, retinoic acid, and caffeine, whereas *Limosilactobacillus* showed a positive correlation with sepiapterin, S-lactoylglutathione, tetrahydrofolyl-[Glu](2), dihydrobiopterin, S-acetyldihydrolipoamide-E, 2-isopropylmalic acid, L-malic acid, and caffeine. Collectively, these three genera were negatively correlated with Xanthosine. In addition, *Ligilactobacillus* showed a significant negative correlation with 4-hydroxynonenal and 7-methylxanthosine. Overall, a significant negative correlation was observed in the FIP group between elevated abundances of *Oscillospirales*, *Helicobacter*, *Roseburia*, *Campylobacter*, and *Enterobacterales*, and reduced levels of metabolites such as S-lactoylglutathione, 2-isopropylmalic acid, 4-amino-4-deoxychorismate, and succinic acid. Additional details are provided in Figure 5E. These correlation patterns are closely linked to FIPV disease progression. The decreased beneficial genera positively correlated with protective metabolites (caffeine derivatives, retinoic acid, folate intermediates) suggest the loss of commensal microbes impairs intestinal immunity and antioxidant capacity after FIPV infection. The expanded pathobionts positively associated with lipid peroxidation marker 4-hydroxynonenal aggravate intestinal oxidative stress and inflammation, which jointly promote the formation of systemic inflammatory lesions in FIP-affected kittens.

## 3. Discussion

**Figure 6:**
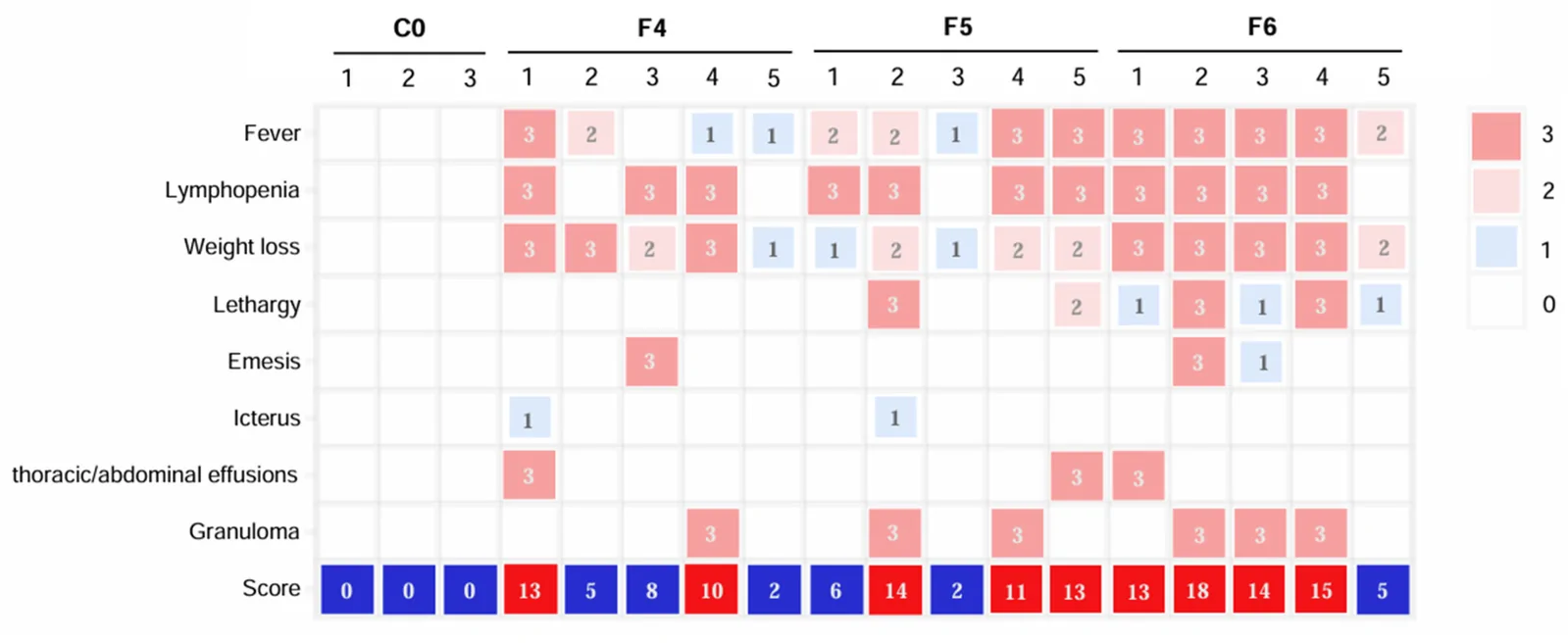
FIPV DF-2 induced clinical disease in experimentally infected kittens. Disease progression was evaluated using an adapted FIP clinical scoring system (Table 3), with parameters summed to generate an overall clinical score per kitten. In the bottom “Score” row, red boxes denote cats with high cumulative clinical scores (severe FIP), blue boxes denote cats with low scores (healthy controls or mildly affected/subclinical cats), and white boxes with black text denote intermediate scores (moderate disease).

In this study, an animal model of feline infectious peritonitis (FIP) was developed by orally inoculating kittens with the DF-2 strain, thereby simulating the natural infection pathway. The establishment of an infection model using this naturally derived strain may more accurately recapitulate the infection route and pathogenic progression that cats encounter during natural FIPV infection [14]. This method of inoculation aligns more closely with the natural progression of FIPV, which involves mutations and systemic dissemination following fecal–oral transmission, as opposed to artificial infection techniques like intraperitoneal injection and intranasal instillation. As a prototypical virulent strain, FIPV DF-2 demonstrates macrophage tropism and stable pathogenicity, effectively inducing both exudative and non-exudative lesions [15]. These characteristics align closely with the typical clinical manifestations of FIP observed in this study, such as increased body temperature, reduced lymphocyte count, ascite formation, and granulomatous lesions. This congruence substantiates the appropriateness of the strain selection and the reliability of the experimental model (Figure 6). Additionally, this research introduces an innovative clinical scoring system for FIP diagnosis (Table 3). This system incorporates a comprehensive array of clinical indicators, facilitating a quantitative assessment of FIP. Unlike previous studies that depended on isolated or incomplete symptom records, this scoring system offers a more systematic and objective evaluation of disease severity, providing standardized criteria for assessment. While the clinical scoring system exhibits promising potential within this model, it is not without limitations. The assignment of weights to scoring indicators is largely informed by existing research experience and lacks statistical validation through large sample data, potentially introducing subjectivity.

Combined with the supplemented RT-qPCR quantification data of anal swabs, we further characterize the features of this oral challenge model. After fecal–oral inoculation, FIPV only transiently replicates in intestinal epithelial cells and is shed via feces at the early infection stage. Once viral mutations occur and disseminate into macrophages to trigger systemic FIP lesions, intestinal viral replication is markedly suppressed, resulting in consistently negative anal swabs in the mid-to-late disease phase. This leads to extreme inter-individual heterogeneity in shedding timing, peak viral load and total shedding duration, without consistent comparable viral replication kinetics across animals. Viral inoculation dose positively regulates early intestinal shedding parameters: the high-dose 6F group exhibited significantly higher peak viral copies, group-positive detection rates, and longer shedding duration than the low-dose 4F group. Nevertheless, fecal viral load could not synchronously reflect the severity of systemic organ lesions nor distinguish effusive and non-effusive FIP phenotypes. The successful establishment of our FIP model was comprehensively validated by cross-verification of composite clinical scores, gross necropsy lesions, histopathological changes, and FIPV N-protein immunohistochemistry. Fecal viral shedding data only serve as supplementary model characterization, rather than the grouping criterion for subsequent fecal microbiome and metabolome differential analysis.

Comprehensive clinical score data illustrated in Figure 6 reveal clear divergence in disease progression across 15 FIPV-challenged kittens. Nine diseased animals developed progressive severe clinical courses with simultaneous aggravation of multiple pathological indicators, whereas six challenged non-diseased kittens only displayed transient mild hyperthermia restricted to early infection, without multi-organ lesions pathognomonic for FIP. This contrast indicates that progression to clinical FIP after oral FIPV exposure is not solely determined by viral inoculation dose, and inter-individual variation in host immune response acts as a critical regulatory factor. Non-diseased kittens in the low-dose group exhibited the mildest clinical perturbations, while the single non-diseased kitten in the high-dose group only suffered brief mild fever. This confirms that higher viral exposure merely elevates morbidity risk rather than inducing sustained clinical abnormalities in resilient individuals. The standardized clinical scoring system clearly distinguishes three phenotypic states: fully developed FIP, transient mild stress post-viral exposure, and complete health, providing objective clinical stratification criteria for subsequent fecal microbiome and metabolome grouping analyses.

The pattern of gut microbial diversity exhibits variability across different diseases. In the present study, a higher microbial species richness in the FIP-affected group (Figure 2A). Furthermore, there is a significant difference in species diversity within the FIP-affected group (Figure 2B). This phenomenon can be attributed to virus-induced dysregulation of immune activation, which suppresses dominant commensal species and leads to the depletion of specific intestinal microbiota. The ensuing transient niche vacancy allows for the expansion of opportunistic pathobionts, paradoxically resulting in increased microbial diversity [16]. In our study, the ratio of *Bacillota* to *Bacteroidota* was reduced in kittens with FIP, which is consistent with previous findings [6]. Notably, the relative abundances of *Bifidobacterium*, *Ligilactobacillus*, and *Limosilactobacillus* were substantially reduced in FIP-affected kittens (Figure 3D). These findings suggest that in the intestines of non-infected kittens, these probiotic taxa may confer host protection, potentially through mechanisms such as competitive exclusion of pathogens and reinforcement of gut barrier integrity [17,18,19]. Existing research indicates that the bacterial genera *Helicobacter*, *Campylobacter*, and *Enterobacterales* represent significant threats to human health, as evidenced by conditions such as *Helicobacter* pylori-associated gastritis, *Campylobacter* jejuni-induced gastroenteritis, and infections caused by multidrug-resistant *Enterobacteriaceae* [20,21,22,23,24,25]. This dysbiotic shift is likely attributable to a synergistic interaction between compromised gut barrier integrity and dysregulated immune responses. Of particular interest are the genera *Oscillospirales* and *Roseburia*, which have been associated with gut health due to their production of the immunomodulatory short-chain fatty acid butyrate [26]. The observed increase in the abundance of *Oscillospirales* and *Roseburia* in the cohort afflicted with FIP may represent a compensatory host response to virus-induced inflammation. However, the precise causative factors and mechanistic underpinnings of this microbial shift require further investigation.

Through non-targeted assay analysis, significant differences in metabolite profiles were identified between the two groups, as detailed in Supplementary Table S3. These differential metabolites are predominantly associated with pathways involved in caffeine metabolism, pyruvate metabolism, folate biosynthesis, hepatocellular carcinoma, non-small cell lung cancer, and Th17 cell differentiation. The disruption of these metabolic pathways may be correlated with the pathogenesis of FIP disease, as illustrated in Figure 5D. The upregulation of 4-hydroxynonenal, 7-methylxanthosine, and xanthosine in fecal samples of cats with FIP is intriguing and warrants further investigation. Additionally, 4-hydroxynonenal, a recognized byproduct of lipid peroxidation, is frequently associated with oxidative stress [27,28]. Its elevated presence in fecal matter may indicate increased oxidative stress within the gastrointestinal tract or reflect systemic oxidative stress manifesting in fecal metabolites. This oxidative stress is potentially a result of the inflammatory response triggered by FIP, as immune activation commonly leads to the generation of reactive oxygen species. Consistent downregulation of caffeine and methylxanthine metabolites was observed in FIP-affected kittens, with uniform caffeine dietary intake across control and infected groups ruling out dietary bias as a driving factor. Such metabolic changes could serve as potential biomarkers, offering deeper insights into the pathological mechanisms underlying FIP and suggesting novel avenues for its diagnosis and treatment.

Notably, the differential metabolite profiles reported herein were rigorously validated by integrating supervised multivariate OPLS-DA modeling and Benjamini–Hochberg FDR multiple comparison correction, which jointly mitigated the risk of false positives originating from the large-scale parallel statistical testing inherent to untargeted metabolomics. The six core perturbed KEGG pathways were exclusively enriched using FDR-verified differential metabolites, and permutation tests were further implemented for pathway enrichment outputs to consolidate the reliability of metabolic signature alterations linked to FIPV infection.

Correlation analyses of gut microbiota and their corresponding metabolomes offer valuable insights into the interactions between the microbiome and gut metabolites. Variations in the abundance of gut flora can influence gut metabolite profiles. Our study identified that *Bifidobacterium*, *Ligilactobacillus*, and *Limosilactobacillus* were positively correlated with significantly downregulated protective metabolites (caffeine metabolites, retinoic acid, folate derivatives) in kittens affected by FIP, whereas opportunistic pathogens such as *Helicobacter*, *Campylobacter*, and *Enterobacterales* were positively correlated with elevated oxidative stress-related metabolite 4-hydroxynonenal. Such microbial–metabolite co-variation reveals that the depletion of beneficial commensals and overgrowth of pro-inflammatory pathobionts synergistically aggravate intestinal barrier injury and systemic inflammatory response, thereby driving the occurrence and progression of FIPV-induced lesions (Figure 5E). These findings highlight the intricate interactions between gut microbial communities and metabolic profiles in cats afflicted with FIP, offering critical implications for the synthesis of biosignaling molecules associated with disease pathogenesis.

Compared with previous related studies, this study fills multiple core gaps in the field, while the limitations of existing studies are mainly reflected in three aspects supported by published studies. First, most earlier studies only performed single 16S rRNA sequencing to describe intestinal bacterial changes after FCoV infection, without integrated untargeted metabolomics to decode microbial–metabolite interactive signatures. Meazzi et al. only analyzed fecal microbiota of naturally FCoV-carried cats rather than kittens with confirmed clinical FIP and failed to explore downstream metabolic disturbances triggered by dysbiosis [6]. Secondly, most existing experimental FIP models have not adopted a graded oral inoculation approach using the DF-2 strain to simulate natural fecal–oral transmission. Instead, they predominantly rely on naturally infected domestic cats, which do not generate graded incidence data that correlate viral exposure intensity with microbial ecological alterations. Consequently, these models are unable to establish dose-dependent relationships between the magnitude of viral challenge and shifts in the gut microbiome [6]. Against these shortages, the uniqueness and innovations of the present work lie in establishing gradient oral FIPV DF-2 kitten model with unified clinical scoring diagnostic criteria; combining 16S microbiome and untargeted fecal metabolomics to screen differential flora and disease-related metabolic pathways; further performing Pearson correlation to interpret pathological relevance of microbe–metabolite co-variation to FIP onset.

For subsequent research directions targeted at the above limitations: (1) Conduct germ-free animal colonization and cell functional assays to verify causal regulatory relationships between core differential genera and characteristic metabolites; (2) Introduce independent parallel healthy control groups and stratified asymptomatic FCoV carriers to distinguish infection-specific microecological alterations from age/stress-induced fluctuations; (3) Combine metagenomics and targeted absolute quantitative metabolomics to mine non-invasive fecal microbe–metabolite biomarkers for early FIP diagnosis, and develop probiotic adjuvant intervention strategies to alleviate FIP systemic inflammation.

## 4. Materials and Methods

### 4.1. Cells and Virus

Crandell–Rees feline kidney (CRFK) cells (Procell, Wuhan, China) were used for viral propagation and titration. Cells were cultured in DMEM (Vivacell, Shanghai, China) supplemented with 10% fetal bovine serum (FBS; ZETA, San Francisco, CA, USA) and 1% penicillin–streptomycin at 37 °C under 5% CO_2_. The FIPV DF-2 strain was purchased from the American Type Culture Collection (ATCC) and passaged three times in CRFK cells. For viral inoculation, cell monolayers were incubated with virus stock diluted 1:100 in serum-free DMEM for 1 h to allow viral attachment. After washing with 1 × PBS, fresh complete DMEM was added for further incubation. At 72 h post-infection, cell cultures were freeze–thawed twice at −80 °C to harvest viral supernatant. Viral titers (TCID_50_) were determined in CRFK cells using the Reed–Muench method [29].

### 4.2. Animals

Eighteen age-matched 6-week-old conventional British Shorthair kittens were purchased from Jianyouda Biomedical Technology Co., Ltd. (Taizhou, Jiangsu, China). Before the experiment, all kittens were screened negative for feline panleukopenia virus (FPV), feline herpesvirus (FHV), feline calicivirus (FCV), and FIPV via virus-specific PCR/RT-PCR on oral and rectal swabs; primer sequences are listed in Supplementary Table S1. Serum neutralization tests further confirmed the absence of antibodies against these four pathogens. All kittens underwent a 21-day acclimatization period prior to viral challenge.

Fifteen kittens assigned to FIPV inoculation groups were housed individually in a negative-pressure laboratory at Yantai Rafael Biotechnology Co., Ltd. The three sham-inoculated control kittens were raised in a separate animal facility. All groups were maintained under identical temperature, humidity, and sanitation conditions, with free access to standard commercial kitten food and water. Throughout the 45-day trial, all animals received identical caffeine-free diets without extra snacks or nutritional supplements to eliminate exogenous caffeine interference.

At the experimental endpoint or when kittens developed terminal FIP symptoms, humane euthanasia was performed as follows: kittens were induced with 5% isoflurane inhalation until unconsciousness. A peripheral venous catheter was placed on the forelimb or hindlimb, and pentobarbital sodium (300–500 mg/mL) was slowly injected intravenously at 100–150 mg/kg to avoid acute discomfort. Respiration and heartbeat were monitored for 5–10 min to confirm complete death before necropsy.

This animal experiment was approved by the Institutional Animal Care and Use Committee of Ludong University on 25 November 2022 (Permit No. LDU-IRB20221125-6). All animal procedures complied with the national laboratory animal welfare standard GB/T 35892–2018 of China.

### 4.3. Virus Challenge

**Figure 7:**
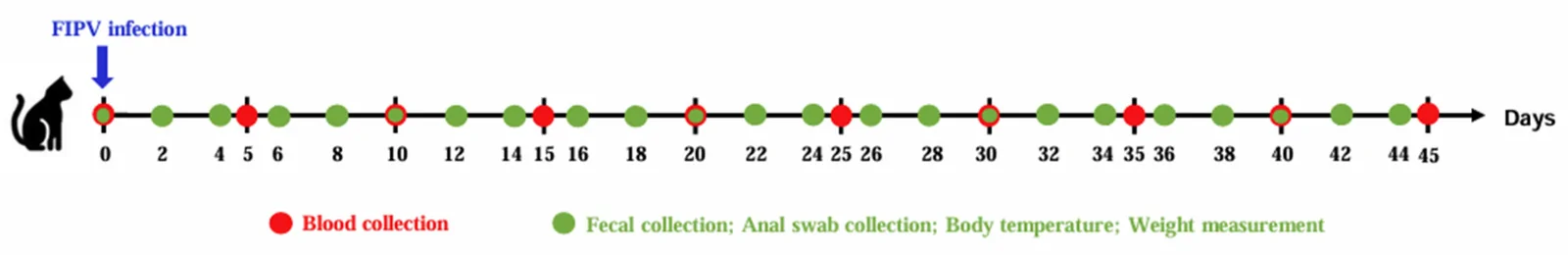
Schematic timeline of experimental procedures over the 45-day study period (0–45 days post-infection, dpi). Fecal samples (*n* = 23 timepoints) and body temperature/weight data were collected at 2-day intervals (0, 2, 4, …, 44 dpi, green points). Venous blood samples (*n* = 10 timepoints) were obtained every 5 days (0, 5, 10, …, 45 dpi, red points).

The 18 kittens were divided into four groups: 5 kittens in the 4F group (1 × 10^4^ TCID_50_ DF-2), 5 in the 5F group (1 × 10^5^ TCID_50_ DF-2), 5 in the 6F group (1 × 10^6^ TCID_50_ DF-2), and 3 uninfected kittens in the C0 control group. The smaller sample size of the control group followed the reduction principle of GB/T 35892-2018: healthy control kittens suffered no FIP-induced lesions or pain, while five replicates per infection group guaranteed sufficient statistical power to capture heterogeneous disease phenotypes for multi-omics comparison. Pre-experimental power calculation verified that three control kittens could provide stable healthy baseline data for omics analysis. The full experimental timeline is shown in Figure 7.

For oral inoculation, viral stocks were diluted in sterile DMEM to final concentrations of 1 × 10^4^, 1 × 10^5^, and 1 × 10^6^ TCID_50_/mL, respectively. Each kitten received 1 mL viral suspension via a 2 mL non-traumatic oral syringe [30,31]. Control kittens were orally administered an equal volume of sterile DMEM as sham inoculation (Table 4).

### 4.4. Clinical Evaluation

**Figure 8:**
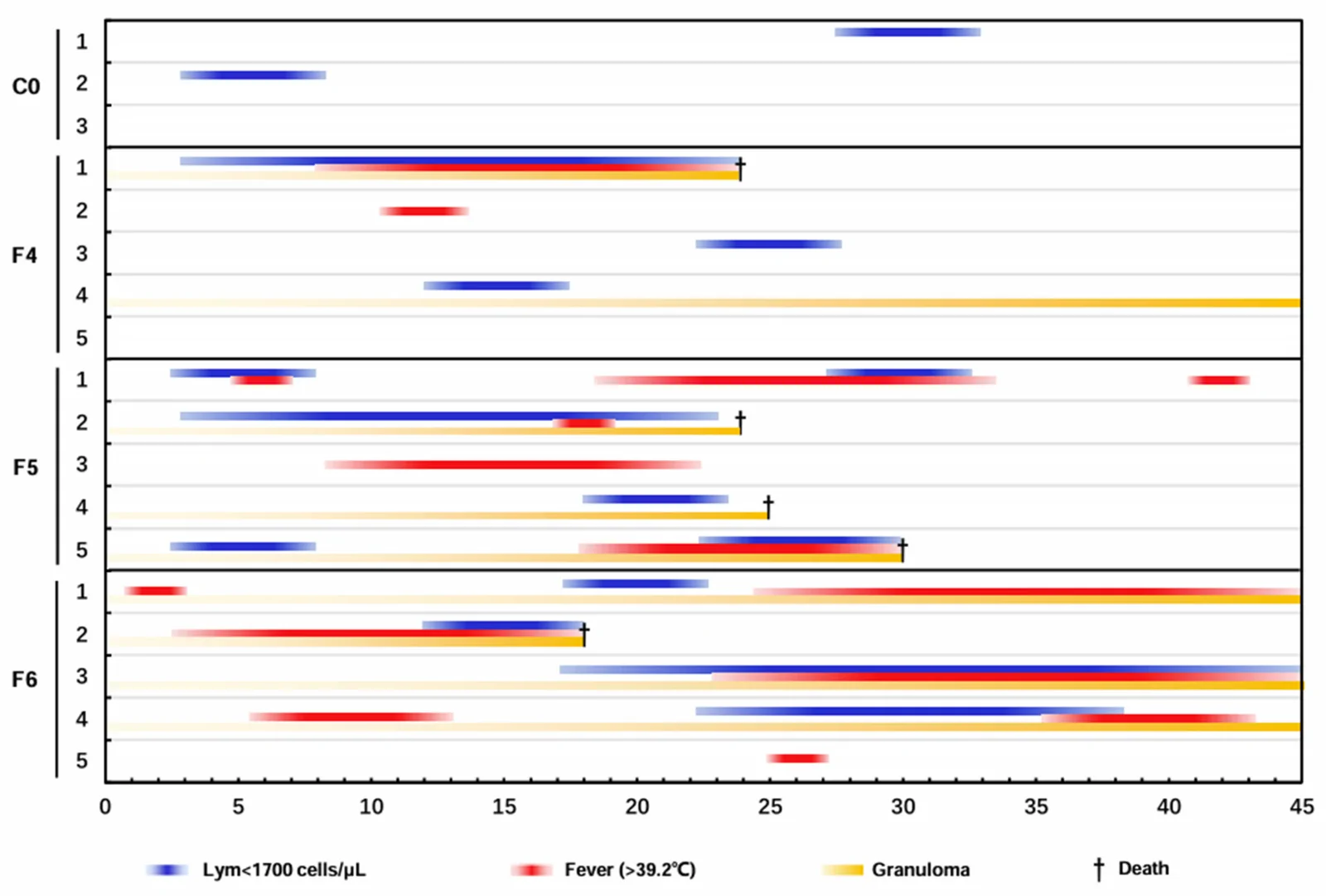
Disease progression timeline in kittens (*n* = 18). Timeline of FIPV-infected symptom onset, fever, lymphopenia, granuloma and death, through the course of disease for 18 kittens.

Animals were monitored daily for the emergence of FIPV-specific clinical signs and indicators of morbidity by a licensed veterinary practitioner. Comprehensive clinical evaluations were conducted at all timepoints using a previously established clinical scoring system [9,32,33]. The full clinical assessment included measurements of body weight, body temperature, activity levels, emesis, icterus, lymphopenia, the presence of ascites or granulomas, and mortality. Each parameter was assigned a score ranging from 0 (normal) to 1–2 (mild–moderate) and 3 (severe), as detailed in Table 2. The individual scores for each clinical parameter were aggregated to provide a cumulative clinical score for each animal over the study period. Kittens exhibiting signs of terminal FIP were humanely euthanized to prevent unnecessary suffering, whereas healthy animals were euthanized at the conclusion of the experiment, followed by a comprehensive post-mortem examination. These cases are documented in detail in Figure 8.

### 4.5. Sample Collection

Fresh fecal and blood samples were systematically collected from all subjects over a period spanning from day 0 to day 45. For each kitten, fresh feces were collected by stimulating the anus with a sterile swab to induce defecation. A fresh fecal sample weighing a minimum of 20 g was obtained every two days and promptly stored at −80 °C. Additionally, to minimize stress in the kittens, 1.5 mL of whole blood was collected from each kitten via cephalic venipuncture every five days and transferred into lithium heparin tubes. In accordance with our laboratory’s standard operating procedures, blood analysis was conducted within four hours post-collection to detect changes in lymphocyte counts. Necropsies were performed on all animals that succumbed or were euthanized during the study. Carcasses were dissected within two hours of death, and any noticeable gross changes in tissues and organs were meticulously documented during the necropsy. For subsequent 16S rRNA amplicon sequencing and untargeted metabolomics detection, balanced paired samples were screened from all collected fecal specimens. Nine fecal samples obtained from clinically confirmed FIP kittens at disease endpoint and nine day-0 pre-inoculation healthy fecal samples were matched as two comparative groups with equal sample size (*n* = 9 per group), and detailed sample matching information is listed in Supplementary Table S2.

### 4.6. DNA Extraction and 16S rRNA Gene Amplicon Sequencing

All stool samples were freshly collected and frozen at −80 °C within 2 h after sampling. DNA was extracted using the FastPure Feces DNA Isolation Kit (YuHua, Shanghai, China) according to manufacturer’s instructions. The concentration and integrity of the extracted DNA were evaluated using a NanoDrop 2000 spectrophotometer (Thermo Scientific, Waltham, MA, USA) and agarose gel electrophoresis, respectively. For library preparation, the hypervariable V3–V4 region of the bacterial 16S rRNA gene was amplified using primer pairs 338F (5′-ACTCCTACGGGAGGCAGCAG-3′) and 806R (5′-GGACTACHVGGGTWTCTAAT-3′) [34]. The PCR conditions were as follows: an initial denaturation at 95 °C for 3 min; 27 cycles of denaturation at 95 °C for 30 s, annealing at 55 °C for 30 s, extension at 72 °C for 45 s; at 72 °C for 10 min; hold at 10 °C. The PCR products were extracted from a 2% agarose gel and purified using the PCR Clean-Up Kit (YuHua, Shanghai, China) according to the manufacturer’s protocol and quantified using a Qubit 4.0 Fluorometer (Thermo Fisher Scientific, Waltham, MA, USA). Purified amplicons were pooled in equimolar amounts and paired-end sequenced on an Illumina PE300 platform (Illumina, San Diego, CA, USA) according to the standard protocols by Majorbio Bio-Pharm Technology Co. Ltd. (Shanghai, China).

### 4.7. Bioinformatic Analysis of 16S-rRNA Gene Sequence Data

Sequence analysis was conducted using UPARSE 7.1. Sequences with ≥97% similarity were allocated to identical OTUs. The most abundant sequence for each OTU was selected as a representative sequence. Prior to analyzing alpha-diversity and beta-diversity, the original OTU table was rarefied to 49,009 reads (the smallest number of bacterial reads among samples) to allow for comparable comparisons. Based on the OTU information, alpha diversity indices including observed OTUs, Chao richness, Shannon index, and Good’s coverage were calculated with Mothur v1.30.1. The similarity among the microbial communities in different samples was determined by principal coordinate analysis (PCoA) based on Bray–Curtis dissimilarity using Vegan v2.5-3 package. The Kruskal–Wallis rank-sum test was used to examine the alterations and disparities between samples. The linear discriminant analysis (LDA) effect size (LEfSe) was performed to identify the significantly abundant taxa (phylum to genus) of bacteria among the different groups.

### 4.8. Untargeted Metabolomics Analysis

#### 4.8.1. Metabolite Extraction

Fecal untargeted metabolomics profiling was performed via UHPLC-MS/MS. Briefly, 50 ± 5 mg fresh fecal sample and one 6 mm grinding bead were transferred into a 2 mL centrifuge. A total of 400 μL extraction solvent (methanol: ultrapure water = 4:1, *v*/*v*) containing 0.02 mg/mL L-2-chlorophenylalanine as internal standard was added for metabolite extraction. Samples were homogenized using a Wonbio-96c cryogenic tissue grinder (Shanghai Wanbo Biotechnology Co., Ltd., Shanghai, China) at −10 °C and 50 Hz for 6 min, followed by low-temperature ultrasonic extraction at 5 °C, 40 kHz for 30 min. The mixture was incubated at −20 °C for 30 min, then centrifuged at 13,000 g, 4 °C for 15 min. The clear supernatant was transferred to glass vials with micro-inserts for instrumental detection. Quality control (QC) samples were prepared by pooling equal 20 μL supernatant aliquots from all individual fecal specimens; QC samples underwent identical extraction and detection procedures to monitor instrumental drift throughout the whole run, following standard untargeted metabolomics sample preparation protocols described previously [35].

#### 4.8.2. UHPLC–MS/MS Detection

All LC-MS detection was completed on a Thermo Vanquish Horizon UHPLC system coupled with an Exploris240 Orbitrap mass spectrometer (Thermo Scientific, Waltham, MA, USA) at Majorbio Bio-Pharm Technology Co., Ltd. (Shanghai, China).

Chromatographic parameters

(1) Analytical column: ACQUITY HSS T3 column (100 mm × 2.1 mm inner diameter, 1.8 μm particle size; Waters, Milford, USA)
(2) Column temperature: 40 °C
(3) Injection volume: 3 μL
(4) Flow rate: 0.4 mL/min
(5) Mobile phase composition: Mobile phase A = 95% LC-MS-grade water + 5% HPLC acetonitrile (0.1% formic acid, *v*/*v*); Mobile phase B = 47.5% acetonitrile + 47.5% isopropanol + 5% water (0.1% formic acid, *v*/*v*)
(6) Linear gradient elution program (total run time 30 min):
(7) 0–3 min: 0–5% B; 3–9 min: 5–35% B; 9–18 min: 35–80% B; 18–24 min: 80–100% B; 24–28 min: hold at 100% B; 28–30 min: re-equilibrate to initial 0% B.
(8) QC injection scheme: One QC sample was inserted every 5–15 experimental injections to assess analytical stability.

Mass spectrometry acquisition parameters

Electrospray ionization (ESI) was operated in both positive (ESI+) and negative (ESI−) modes for parallel metabolite capture:

(1) Full scan mass range: *m*/*z* 70–1050
(2) Full MS resolution (*m*/*z* 200): 60,000
(3) MS/MS secondary fragmentation resolution (*m*/*z* 200): 15,000
(4) Normalized stepped collision energy (NCE): 20%, 40%, 60%
(5) Ion source gas settings: Sheath gas = 60 arb; Auxiliary gas = 20 arb
(6) Temperature settings: Ion heater = 350 °C; Capillary = 320 °C
(7) Spray voltage: ESI+ = +3400 V; ESI− = −3000 V
(8) S-Lens RF level: 70

The UHPLC-Orbitrap instrumental configuration applied here follows validated analytical workflows for mammalian gut metabolome and lipid profiling [35].

#### 4.8.3. Data Preprocessing & Comprehensive Lipid/Metabolite Database Search Parameters

Raw LC-MS/MS spectra were processed in Progenesis QI v3.0 (Waters, USA) for baseline correction, peak picking, peak integration, retention time alignment, and cross-sample feature matching. Pre-filtering rules before library matching:

Only metabolic features detected in ≥80% of samples within each group were retained; Missing peak intensity values were filled with the minimum intensity of the corresponding feature across all samples; All peak areas were normalized to total ion current (TIC); Features with relative standard deviation (RSD) > 30% across all QC samples were discarded to exclude unstable signals; Remaining feature intensities were log10-transformed for subsequent multivariate statistics.

Metabolite and lipid annotation was performed against three databases: HMDB, Metlin, and the in-house Majorbio Database (MJDB, including a dedicated Lipidmaps lipid sub-library). Uniform library matching thresholds applied to all datasets:

Precursor ion mass tolerance: ≤ 10 ppm; Fragment ion mass tolerance: ≤0.02 Da;

Adduct ion inclusion list: ESI+ ([M+H]^+^, [M+Na]^+^, [M+NH4]^+^); ESI− ([M-H]^−^, [M+FA-H]^−^, [M+Cl]^−^); Lipid identification criteria: Valid lipid annotation required consistent precursor *m*/*z* matching plus ≥2 diagnostic MS/MS fragment ions unique to lipid subclasses; Statistical correction: Benjamini–Hochberg false discovery rate (FDR) correction was applied to all matched features; only metabolites with FDR-adjusted *p* < 0.05 were considered credible annotations for downstream pathway enrichment. Lipid identification and database matching criteria conformed to established lipidomic mass spectrometry identification standards [7,8], and oxidative lipid metabolite analytical pipelines [27].

Multivariate statistical modeling was conducted via the R ropls package (v1.6.2), including PCA and orthogonal partial least squares discriminant analysis (OPLS-DA). Seven-round permutation testing was used to validate model robustness and eliminate overfitting. Differential metabolites were defined by dual screening criteria: OPLS-DA variable importance in projection (VIP) > 1 + Student’s t-test raw *p* < 0.05, with FDR correction for multiple testing. Significantly altered metabolites were mapped to the KEGG database for enrichment analysis using Python (v3.9.7) scipy.stats functions.

## 5. Conclusions

In conclusion, this pilot discovery study preliminarily screened distinct candidate gut microbial and metabolic alterations that correlate with FIP status in orally challenged kittens using self-baseline samples as controls. Notably, the relative abundances of *Ligilactobacillus*, *Limosilactobacillus*, and *Bifidobacterium* were significantly diminished (*p* < 0.05) in FIP-affected kittens, whereas the relative abundances of *Helicobacter*, *Campylobacter*, and *Enterobacterales* were significantly elevated (*p* < 0.05). Furthermore, metabolomic analysis revealed differential metabolites and associated metabolic pathways, including caffeine metabolism, pyruvate metabolism, folate biosynthesis, hepatocellular carcinoma, non-small cell lung cancer, and Th17 cell differentiation, as critical pathways linked to the dysbiosis of fecal microbiota in FIPV-infected kittens.

## 6. Limitations of the Study Design

This study has a key experimental design limitation: no separate concurrent age-matched uninfected negative control group was established throughout the trial. We used day-0 pre-inoculation fecal samples from each kitten as longitudinal self-controls to balance omics sample sizes. While self-baseline samples reduce individual biological variation, they cannot fully eliminate confounding effects from kitten age growth, prolonged laboratory rearing stress and time-dependent gut microbial natural shifts over the 45-day trial. Without parallel uninfected animals sampled at identical timepoints, we cannot quantitatively correct age/time-induced microbial and metabolic fluctuations. Thus, consistent with the positioning of this work as a pilot discovery study, all observed fecal microbe–metabolite differences only reflect statistical correlation with FIPV infection and disease onset, and we cannot fully attribute these alterations solely to viral pathogenesis without parallel age-matched uninfected controls. Follow-up research with synchronized uninfected control kittens is required to separate age-related interferences and validate our candidate FIP-associated microbial–metabolic signatures.

## Figures and Tables

**Table 1:** FIP infection rates and ID_50_ calculation of FIPV DF-2 strain.

Virus Dilution	Observed Values			Cumulative Values		
	Positive	Negative	% Infection	Positive	Negative	% Infection
1 × 10^6^	4	1	80	9	1	90
1 × 10^5^	3	2	60	5	3	62.5
1 × 10^4^	2	3	40	2	6	25

**Table 2:** Enriched KEGG pathways and their corresponding differential metabolites.

KEGG Pathway Description	Metabolite
Caffeine metabolism	Caffeine; 1-methyluric acid; 7-Methylxanthosine; 1-Methylxanthine; 7-Methylxanthine; Xanthosine
Pyruvate metabolism	S-Acetyldihydrolipoamide-E; 2-isopropylmalic acid; S-Lactoylglutathione; Succinic acid; L-malic acid
Folate biosynthesis	4-amino-4-deoxychorismate; Dihydrobiopterin; Sepiapterin; Tetrahydrofolyl-[Glu](n); Tetrahydrofolyl-[Glu](2)
Hepatocellular carcinoma	4-Hydroxynonenal; Dg(11d3/9d3/0:0)
Non-small cell lung cancer	Dg(11d3/9d3/0:0); Retinoic acid
Th17 cell differentiation	Dg(11d3/9d3/0:0); Retinoic acid

**Table 3:** Clinical Scoring System for FIP.

Clinical Parameter	Score			
	0 (Healthy)	1	2	3
Temperature(°C)	37.2 ≤ T < 39.2	39.2 ≤ T < 39.5	39.5 ≤ T < 39.7	≥39.7 °C
Lymphopenia	None	N/A	N/A	≤1700 cells/μL
Weight loss	No weight loss	0 to 5% weight loss	5 to 10% weight loss	>10% weight loss
Activity	Normal	Mild reduction when disturbed (mild lethargy)	Moderate reduction when disturbed (moderate lethargy)	Little to no activity disturbed and reduced activity when stimulated
Emesis	None	Occasional, rare vomiting	Intermittent vomiting (one episode everyday)	Marked persistent vomiting (two or more episodes everyday)
Icterus	None	present	N/A	N/A
thoracic/abdominal effusions	None	N/A	N/A	present
Granuloma	None	N/A	N/A	present

Table legend: Clinical indicators were graded on a scale ranging from 0 (no abnormality) to 3 (severe pathognomonic lesions of FIP). A cumulative total clinical score above 10 was set as the diagnostic cutoff for FIP onset. Score 1 represents transient mild abnormalities, Score 2 indicates persistent moderate clinical signs, and Score 3 corresponds to severe hallmark lesions including lymphopenia (≤1700 cells/μL), thoracic/abdominal effusions, or visceral granulomas.

**Table 4:** Experimental Cat Groupings and Infection Doses.

Group	Infection Dose	Infection Route	Animal Count
C0	DMEM, 1 mL	oral	3
4F	1 × 10^4^ TCID_50_/mL, 1 mL	oral	5
5F	1 × 10^5^ TCID_50_/mL, 1 mL	oral	5
6F	1 × 10^6^ TCID_50_/mL, 1 mL	oral	5

## Data Availability

The raw data supporting the conclusions of this article will be made available by the authors on request. 16S rRNA gene data has been presented in the National Center for Biotechnology Information (NCBI) Sequence Read Archive (SRA) database under accession number: PRJNA1346392; Metabolomics raw data have been deposited in MetaboLights: MTBLS13202.

## Supplementary Materials

The following supporting information can be downloaded at: https://www.mdpi.com/article/10.3390/ijms27177843/s1.
